# No Evidence for Reproductive Isolation through Sexual Conflict in the Bulb Mite *Rhizoglyphus robini*

**DOI:** 10.1371/journal.pone.0074971

**Published:** 2013-09-19

**Authors:** Agata Plesnar-Bielak, Anna M. Skrzynecka, Zofia M. Prokop, Michał Kolasa, Maciej Działo, Jacek Radwan

**Affiliations:** 1 Institute of Environmental Sciences, Jagiellonian University, Kraków, Poland; 2 Institute of Environmental Biology, Faculty of Biology, Adam Mickiewicz University, Poznań, Poland; University of Arkansas, United States of America

## Abstract

Sexual conflict leading to sexual antagonistic coevolution has been hypothesized to drive reproductive isolation in allopatric populations and hence lead to speciation. However, the generality of this speciation mechanism is under debate. We used experimental evolution in the bulb mite *Rhizoglyphusrobini* to investigate whether sexual conflict promotes reproductive isolation measured comprehensively to include all possible pre- and post-zygotic mechanisms. We established replicate populations in which we either enforced monogamy, and hence removed sexual conflict by making male and female evolutionary interests congruent, or allowed promiscuity. After 35 and 45 generations of experimental evolution, we found no evidence of reproductive isolation between the populations in any of the mating systems. Our results indicate that sexual conflict does not necessarily drive fast reproductive isolation and it may not be a ubiquitous mechanism leading to speciation.

## Introduction

Sexual selection and sexual conflict are thought to be important evolutionary forces facilitating speciation (reviewed by Ritchie [1]). For example, run-away sexual selection [2,3,4] can lead to divergence in mating preferences between populations (reviewed in Coyne and Orr [5]). More recently, the role of sexual conflict in causing reproductive isolation has been emphasized (reviewed in Gavrilets and Hayashi [6]).

Sexual conflict can arise in promiscuous species, because fitness optima for traits connected with reproduction are expected to differ between males and females [7]. Selection acting on males should favor adaptations that help to coerce females into mating [8], prevent them from remating with subsequent partners [9] and increase a male’s chance of success during sperm competition with a female’s other mates [10]. If such adaptations in males reduce the lifetime reproductive success of their partners [7,11,9,12,8], females should be selected for counter-adaptations that would increase their resistance to such male manipulations [9,13,14,15]. As female counter-adaptations, in turn, hamper male reproductive success and hence their fitness, males are expected to evolve traits overpowering female defenses. This process is called sexually antagonistic coevolution and can be described as an evolutionary arms race between the sexes [7,16,8].

Sexual conflict has been hypothesized to play an important role in speciation [17]. If allopatric populations (isolated populations with no contact and no gene flow) evolve diverging adaptations in the process of sexually antagonistic evolution, reproductive isolation between them may arise rapidly, facilitating further divergence through genetic drift or local adaptation [18,19,20]. A study on dung flies *Sepsis cynipsea* suggests that reproductive isolation may indeed occur after a very short time when sexual conflict is operating [21]. However, similar experiments on *Drosophila melanogaster* [22], *D* *. pseudoobscura* [23], *Callosobruchus* *maculatus* [24] and *Tribolium* *castaneum* [25] failed to find evidence for reproductive isolation arising through sexual conflict. Hence, it remains unclear how prevalent is the phenomenon of rapid reproductive isolation as a result of sexual conflict.

Here, we used experimental evolution in the bulb mite *Rhizoglyphusrobini* (Acari, Acaridae) to test if sexual conflict promotes reproductive isolation under allopatry. *R* *. robini* is a highly promiscuous species in which females seem to mate indiscriminately, but intra-sexual selection is strong both pre-copulation, including mortal combats between males, and post-copulation (reviewed in 26). Sexual conflict in this species has been observed for mating frequency (excessive mating frequency hampers female lifetime reproductive success [27]) and male sperm competitiveness (females suffer reduced fecundity when mated with males carrying an allele increasing fertilization success [28]). The potential for sexually antagonistic coevolution has been confirmed in this species using an experimental evolution approach. Specifically, males from lines evolving under enforced monogamy became less harmful to females, at the cost of reduced reproductive competitiveness in comparison to males from lines in which the naturally occurring polygamous mating system had been retained. Also, females from monogamous lines evolved reduced resistance to male harm [29].

We established replicate populations in which we either enforced monogamy (20 individually isolated pairs per population) or allowed promiscuity (20 males and 20 females interacting freely). While sexual conflict was operating in promiscuous populations, it was removed from the monogamous ones, as 100% monogamy leads to compatibility of the evolutionary interests of males and females [30]. After 35 and 45 generations of evolution, we measured reproductive isolation between populations evolving under the same regime.

We measured pre-zygotic reproductive isolation associated with male-male competition and female choice, by checking whether males are successful in competition for females from their own population against rivals from “allopatric” populations. Such a difference could thus arise because females are more eager to mate with “sympatric” than with “allopatric” males and/or because males are better in competition for “sympatric mates”. For example, sexually antagonistic coevolution of male harm associated with high sperm competitiveness and female resistance [29] occurring independently in isolated populations may lead to males being more efficient than “allopatric” males in fertilizing females from their own population.

We also used a composite measure of reproductive isolation consisting of male mate choice and post-zygotic isolation by checking whether males housed simultaneously with females from their own population and from an “allopatric” population produce more offspring with the former. More offspring produced by “sympatric” females could be due to post-zygotic isolation mechanisms connected with embryonic and early larval viability, but could also be due to higher likelihood that the “allopatric” female will not mate, for example because “sympatric” females are more attractive or simply easier to detect. Thus, our two composite measures included all possible sources of reproductive isolation we could envision.

## Materials and Methods

### General procedures

Base populations and larger groups of mites were maintained in plastic containers (2.5 cm in diameter and 2 cm high), whereas individuals and small groups of mites were kept in 0.8 cm diameter glass tubes (2 cm high) with plaster of Paris bases soaked with water. Mites were maintained at 24°C, under humidity of >90% and fed powdered yeast *ad libitum*.

### Base population

The mites used in the experiment originated from a stock culture combined of two populations derived from colonies of ca. 200 individuals found on onions in 1998 and 2008 in a garden near Kraków, Poland. Since collection from the field, each population had been maintained in the lab at large numbers (>1000 individuals). The two populations had been mixed approximately ten generations prior to the beginning of experimental evolution. This was done to increase genetic variation which is crucial in laboratory experimental evolution where adaptations arise from standing genetic variation rather than *de novo* mutations, because of the limited time span and population sizes (reviewed in Barrett & Schulter [31]).

### Promiscuous and monogamous lines

To test the impact of mating system on the evolution of reproductive barriers, we established five promiscuous lines in which sexual conflict was operating and five monogamous lines where sexual conflict was largely removed, since full monogamy makes female and male evolutionary interests congruent.

For monogamous lines during each generation, we randomly assigned 20 pairs of mites and allowed them to interact for 5 days, so that multiple mating with the same partner was possible. In promiscuous lines, 20 males and 20 females were placed into one container, thus enabling male competition for access to females. After five days, all females within each line, whether monogamous or polygamous, were transferred to a common container to lay eggs. Densities of ovipositing females and developing larvae were low in all the lines, which was ensured by the size of a container. After ca. 10 days, when tritonymphs (the last larval stage in bulb mites) emerged, about 80-90 individuals were isolated to separate glass tubes. Emerging adults were then sexed and 20 individuals of each sex from each line were used to start a new generation. Reproductive isolation assays (see below) were carried out after 35 (post-zygotic isolation index) and 45 (pre-zygotic isolation index) generations of the experimental evolution.

### Reproductive isolation assay 1: pre-zygotic mechanisms under male competition

We established groups of three individuals (4-6 days old), each group consisting of one female, one male from the same line (“sympatric” male) and one male from another line within the same mating system (“allopatric” male). The bulb mite is a male dimorphic species, with aggressive fighter males capable of killing other males coexisting with benign scrambler males [32]. Although the morphs do not differ in sperm competitiveness [33], whenever it was possible we used males of the same morph in each group. In seven replicates (groups) males were not matched (two in promiscuity and five in monogamy treatments). We also discarded from analysis the replicates in which one of the males had been killed during the experiments (2.5% of all trials), because in such cases the outcome of intrasexual competition could not reveal reproductive isolation between lines as it depended little, if at all, on pre- or post-copulatory male-female interactions.

**Figure 1:**
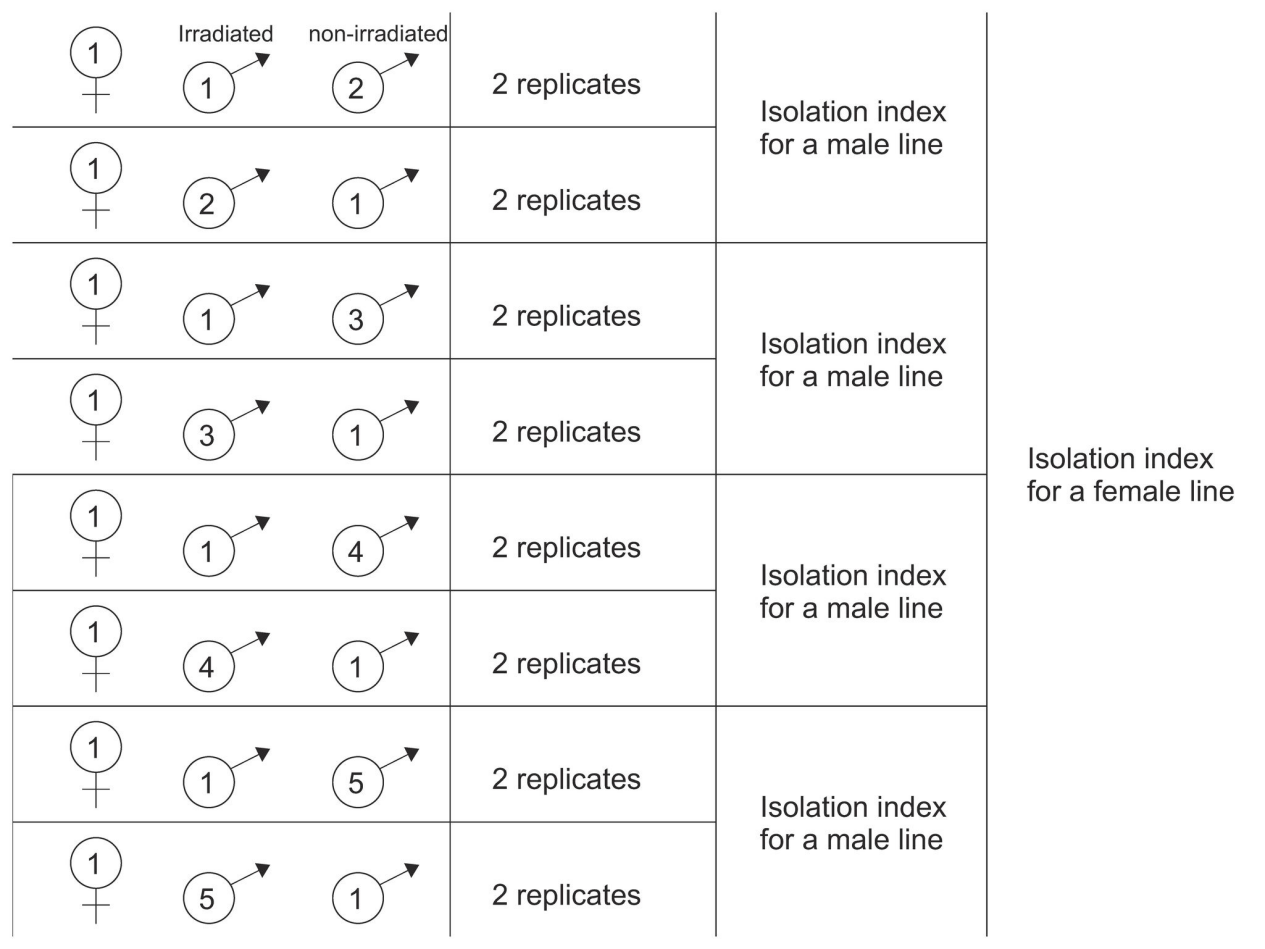
Schematic representation of the design of Assay I. Schematic representation of the design of Assay I and the way of calculating pre-zygotic isolation mechanisms under male competition index on the example of a female’s line 1. Procedures are described in the text.

In order to determine paternity, one of the two males in each group was sterilized using 20kRad (200Gy) gamma radiation from cobalt-60. Males treated this way produce viable sperm, but no eggs fertilized with this sperm hatch [33]. As natural egg viability is >98% [34], the proportion of unhatched eggs is a good proxy of a proportion of eggs fertilized by an irradiated male. Four replicates (groups) were established for each female line–male line combination; in two replicates “sympatric” male was irradiated whereas in the other two, “allopatric” male was irradiated (Figure 1). If one of the replicates within a female line–male line combination had to be excluded from the analyses (because a female died or produced less than 10 offspring or one of the males had been killed) we randomly excluded another replicate, so as to ensure equal contribution of irradiated and non-irradiated “allopatric” males to calculating isolation index for each line combination. Such balanced design should prevent any irradiation-mediated decline in male fertilization ability from influencing the measures of reproductive isolation obtained from this experiment.

The mites in each replicate were left to interact freely for about 24 hours, during which the males had the ability to compete for the access to female gametes. Subsequently, the males were discarded and the females were left to lay eggs for another three days, and then removed. Seven days later we counted all eggs and larvae in each mating vial. We calculated the isolation index for each female line – “allopatric” male line combination as a mean proportion of eggs fertilized by “allopatric” in competition with “sympatric” males, and isolation index for each female line as a mean of the indices for the four “allopatric” lines (see Figure 1).

An index value close to 0 indicates strong reproductive isolation. An index value of 0.5 is a random expectation: neither “sympatric” nor “allopatric” males have higher fertilization success in our lines (no reproductive isolation). To check whether reproductive isolation between replicate lines evolved within either of our mating system treatments, we used one-sample t tests with mean indices for female lines as units of observation.

### Reproductive isolation assay II – male mate choice and post-zygotic reproductive isolation

**Figure 2:**
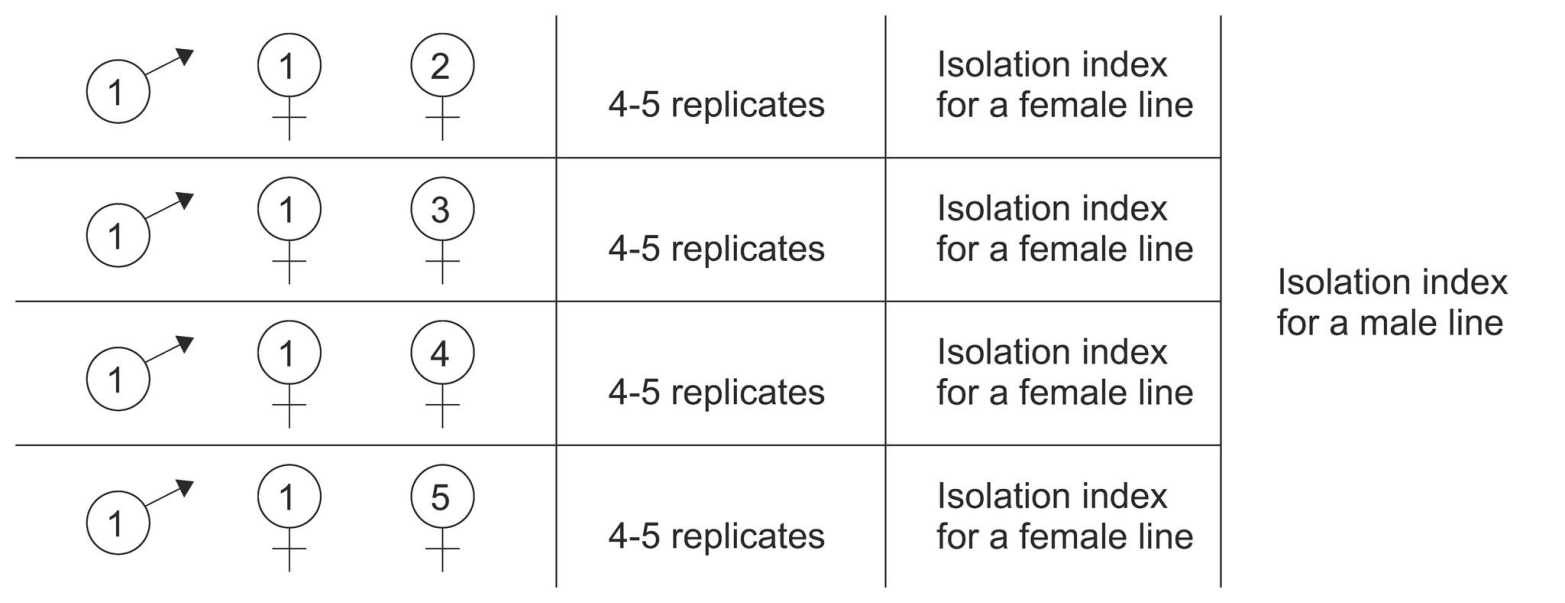
Schematic representation of the design of Assay II. Schematic representation of the design of Assay II and the way of calculating mate choice and post-zygotic reproductive isolation on the example of a male’s line 1. Procedures are described in the text.

We established groups of three individuals, each group consisting of one male, one female from the same line (“sympatric” female) and one female from another line within the same mating system (“allopatric” female). Between 16 and 20 males per line were used to form such groups, 4-5 for each of the four “allopatric” female lines (Figure 2). Hence, all possible line combinations were established within each mating system.

Each group was kept together for 4 hours, after which the males were discarded and females were placed in individual glass tubes to lay eggs. After 14 days, the numbers of offspring produced by each female were counted. For each female line-male line combination (see Figure 2), we calculated an index I, the total number of offspring produced by the “allopatric” females from a given line divided by the total number of offspring produced by all females from this line. We then calculated the isolation index for each male line as a mean of those indexes (Figure 2).

The assay was run using 2-4 days old virgin individuals. In order to distinguish between “sympatric” and “allopatric” females, half of them were dyed using Nile Blue dye. To be sure that female coloration did not influence our results we additionally ran a separate analysis for those replicates in which an allopatric female either had or had not been dyed.

The closer to 0 the index is, the stronger the isolation. An index value of 0.5 indicates that matings with “sympatric” and “allopatric” females produce the same number of offspring (no isolation). We used one-sample t tests to check whether reproductive isolation between replicate lines evolved within either of our mating system treatments.

### Comparison between mating systems

The isolation indices were compared between mating systems using Student’s t tests, with mean female lines’ indices (Assay I) and male lines’ indices (Assay II) as units of observation. If sexual conflict facilitates reproductive isolation, we would expect the isolation indices to be significantly lower in the promiscuous treatment.

### Ethics statement

The mites used in the experiment have been collected in a garden near Kraków, Poland. The owner of the land gave permission to conduct the study on this site. No specific permissions were required as the study did not involve endangered or protected species. According to Polish law specific permissions are required only for studies involving vertebrates or endangered or protected species.

## Results

**Figure 3:**
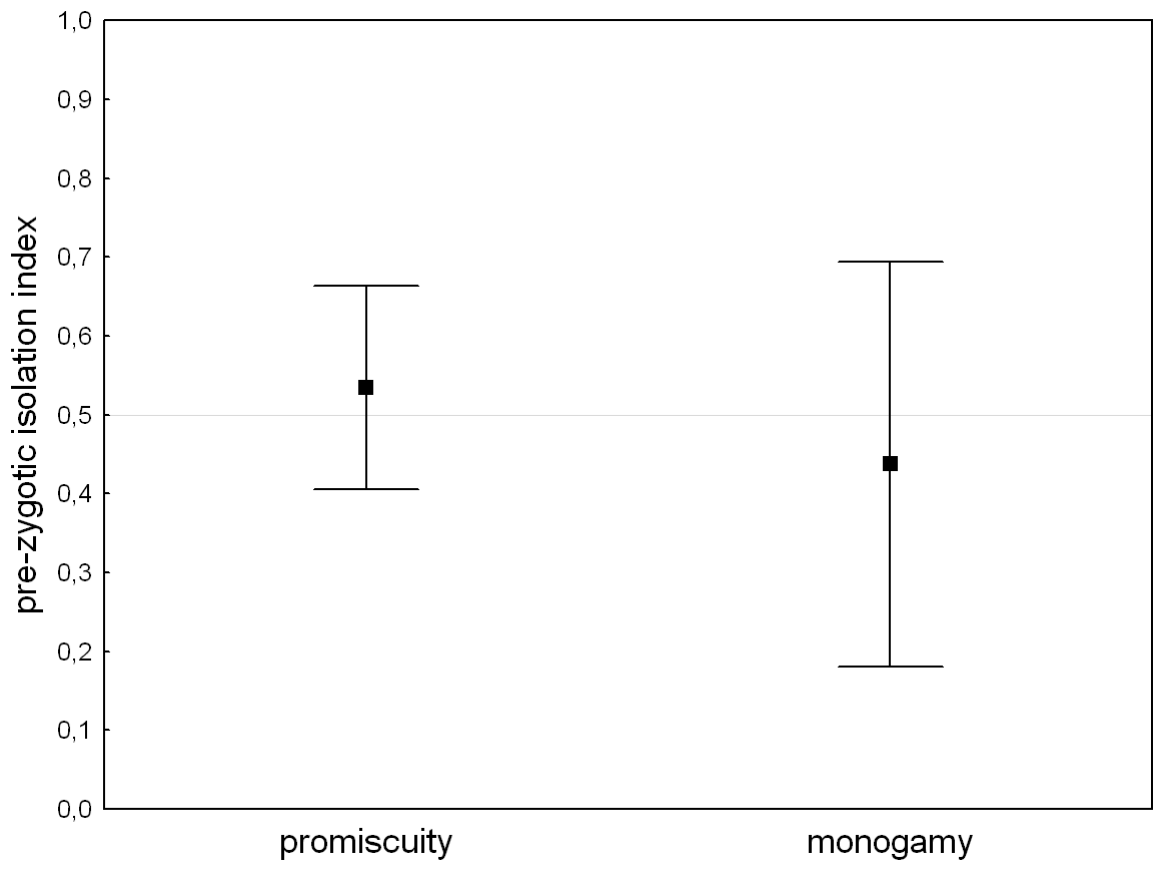
Pre-zygotic isolation indexes measured under male competition for monogamous and promiscuous mating system. Pre-zygotic isolation indices measured under male competition for monogamous and promiscuous mating system calculated from the mean indices for female lines. Indices values lower than 0.5 indicate that a male from the same line as a female fertilizes on average more eggs than a rival from different line. Bars indicate 95% confidence intervals.

**Figure 4:**
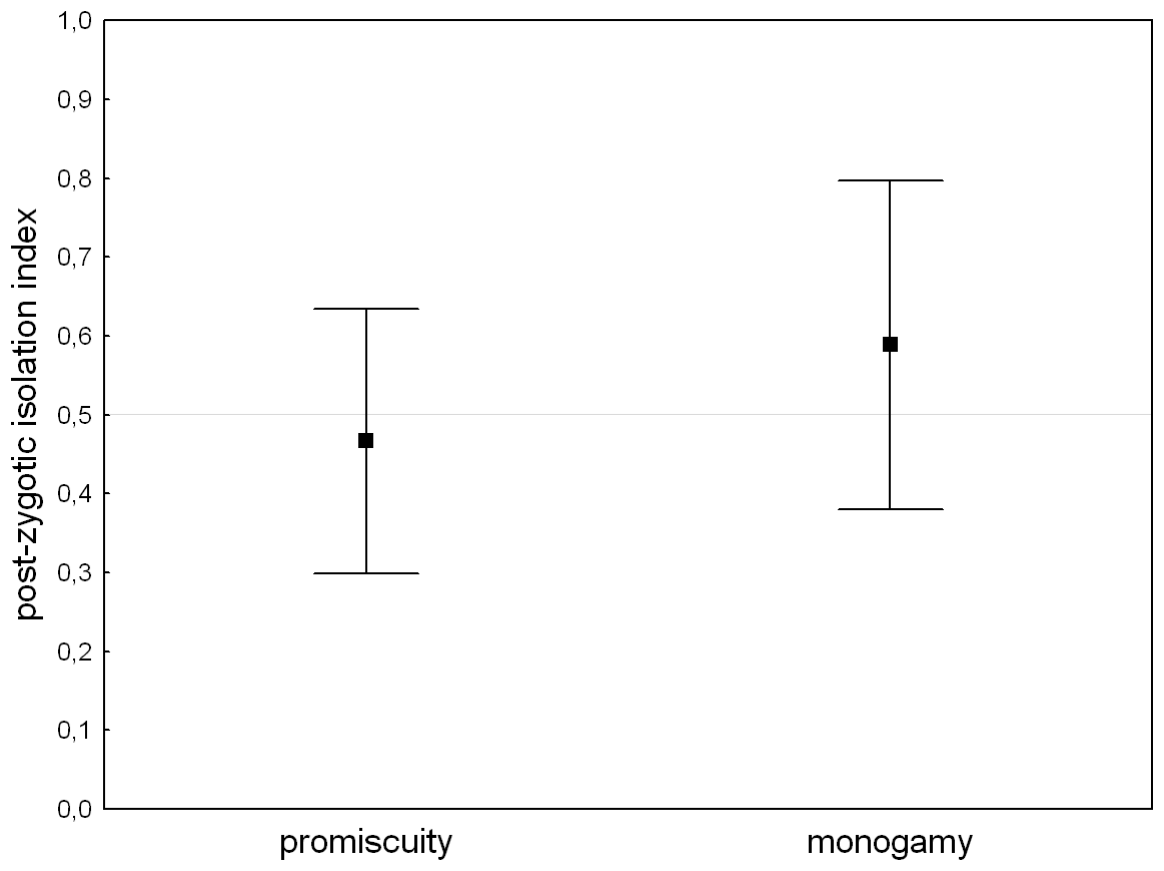
Mate choice and post-zygotic isolation indexes for monogamous and promiscuous mating system. Mean mate choice and post-zygotic isolation indices for monogamous and promiscuous mating system calculated from the mean indices for male lines. Indices values lower than 0.5 indicate that a female from the same line as a male produces on average more offspring than a female from a different line. Bars indicate 95% confidence intervals.

We did not find any evidence for reproductive isolation within either mating system: mean isolation indices in assay I (monogamy: t_4_=-0.679, P=0.534; promiscuity: t_4_=0.752, P=0.494), as well as in assay II (monogamy: t_4_=1.180, P=0.303 promiscuity: t_4_=-0.549, P=0.612) did not differ significantly from 0.5 (Figures 3 & 4). Mating systems did not differ in any of isolation indices (assay I: t_8_=0.944, P=0.374, assay II: t_8_=-1.264, P=0.243). Performing separate analysis for replicates where the “allopatric” individuals were dyed and for those where the “sympatric” individuals were dyed gave qualitatively identical results.

## Discussion

Theoretical models predict that sexually antagonistic coevolution, driven by sexual conflict, may facilitate reproductive isolation and speciation [17,18,6]. However, after 35 and 45 generations of experimental evolution, we found no evidence for reproductive isolation between either promiscuous or monogamous populations of *Rhizoglyphusrobini* in two comprehensive tests involving many possible mechanisms of reproductive isolation (female and male choice, post-copulatory sexual selection and post-zygotic isolation).

Our results are in strong contrast with those obtained by Martin and Hosken [21] who observed occurrence of sexual isolation in dung fly populations after 35 generations of evolution under sexual conflict (compared to no isolation between monogamous populations). Although levels of isolation were higher among larger populations, reproductive isolation was also highly significant among promiscuous populations of similar sizes to those maintained in our study. Population sizes, experimental design and number of generations after which the isolation was tested, were thus comparable among the studies and are unlikely to be the cause of the contrasting outcomes.

According to theoretical models, there are several factors affecting the chances that sexual conflict will drive reproductive isolation. Parker and Partridge [17] suggest that for reproductive isolation to evolve, females must have an “upper hand” in the conflict for mating rate. Mating frequency in bulb mites is quite high, with mean remating time of about 80 minutes [34], even though multiple mating decreases female lifetime fecundity, as shown by Kołodziejczyk & Radwan [27]. This indicates that females in this species do not mate at the optimal rate. Moreover, when males invest little in mating (which seems to be the case in the bulb mite), it is unlikely for them to be highly discriminative, which should slow down the evolution of reproductive isolation [17,19].

In addition, Gavrilets and Hayashi [6] describe at least six types of evolutionary dynamics of sexual conflict, only one of which is expected to drive reproductive isolation under allopatry. Rapid evolution of reproductive barriers may occur if sexual conflict takes the form of a continuous evolutionary chase (arms race). However, if the costs of male and female sexually antagonistic traits are incorporated into the model, sexually antagonistic coevolution is not perpetual – it eventually leads to an equilibrium [35,36] or a stable limit cycle [35]. Hence, if male and female traits involved in sexual conflict are costly, the conflict should be expected to favor reproductive isolation only during a time-limited “arms race” phase before the equilibrium (or limit cycle) is reached, or after a population has been displaced from it.

Our experimental evolution lines were indeed displaced from the equilibrium state which might have evolved in the stock population. The lines experienced 1:1 sex ratio at the outset of each generation, whereas in our base colony the sex ratio is highly female biased (ca. 1:2.4, unpublished), due to fight-related male mortality and slightly female-biased primary sex ratio. Thus, sexual conflict in our promiscuous lines was more intense than in the stock culture, but this did not result in reproductive isolation. Similarly, Wigby and Chapman [22], Bacigalupe et al. [23] and Michalczyk [25] failed to find reproductive isolation in *D. melanogaster*, *D* *. pseudoobscura* , and *T* *. castaneum* (respectively), despite significantly altering the level of sexual conflict (see Table 1). In particular, Bacigalupe et al. [23] investigated several measures of both pre- and post-mating isolation and still did not find any evidence for isolation through sexual conflict. In fact, some of their outcomes contradicted predictions of this hypothesis as populations evolving at high levels of conflict had lower mating latency when mated in allopatry than in sympatry (see Table 1). Also, Gay et al. [24] did not find any evidence of reproductive isolation between replicate populations in which sexual conflict was reintroduced after 90 generations of evolution under monogamy (no conflict) (Table 1). However, all the studies measured reproductive isolation after a relatively short time (up to 50 generations, see Table 1) and thus it might have not been enough time for sexual conflict to accumulate changes in the populations so that isolation would be detectible. On the other hand, sexual conflict is believed to drive reproductive isolation rapidly.

**Table 1:** Experimental evolution studies testing the hypothesis that sexual conflict drives reproductive isolation in allopatry.

Reference	Species	Gs.*	Treatments ^†^	Variables measured	Results
Martin & Hosken 2003	*S* *. cynipsea*	35	1. monogamy (20 pairs per line) 2. low density promiscuity (25 ♀ + 25 ♂ per line) 3. high density promiscuity (250 ♀ + 250 ♂ per line)	1. Proportion of pairs mating 2. Proportion of ♀ showing no reluctance to mate; both variables measured for sympatric^‡^ and allopatric^‡^ crosses.	Treatments: both proportions much lower for allopatric than for sympatric crosses. Monogamy: no differences between allopatric and sympatric crosses.
Wigby & Chapman 2006	*D. melanogaster*	41	1. ♀ biased sex ratio (75 ♀ + 25 ♂ per line) 2. Equal sex ratio (50 ♀ + 50 ♂ per line) 3. ♂-biased sex ratio (25 ♀ + 75 ♂ per line)	Proportion of pairs mating; measured for sympatric and allopatric crosses.	No significant differences between allopatric and sympatric crosses in any of the treatments.
Bacigalupe et al. 2007	*D* *. pseudoobscura*	50	1. monogamy (80 pairs per line) 2. polygamy (40 × (1 ♀ + 3 ♂) per line) 3. elevated polygamy (40 × (1 ♀ + 6 ♂) per line)	1. Proportion of failed matings 2. Mating latency 3. Mating duration 4. F1 ♂ inviability 5. F1 ♂sterility; measured for sympatric and allopatric crosses.	No significant differences in proportion of failed matings between allopatric and sympatric crosses in any treatment. Shorter mating latency in allopatric than in sympatric crosses in elevated polygamy treatment^§^,no difference in other treatments. No significant influence of cross type on copulation duration in any treatment. Viable F1 ♂ offspring produced in all allopatric and sympatric matings. No influence of cross type on F1 ♂ sterility in any treatment.
Michalczyk 2008	*T* *. castaneum*	20	1. ♀ biased sex ratio (90 ♀ + 10 ♂ per line) 2. ♂-biased sex ratio (15 ♀ + 90 ♂ per line)	Offspring number; measured for sympatric and allopatric crosses.	No significant differences between allopatric and sympatric crosses in either treatment.
Gay et al. 2009	*C* *. maculatus*	19	1. Small populations (50 individuals) 2. Large populations (500 individuals); all promiscuous but derived from lines maintained for 90 generations under monogamy	1. Proportion of failed matings 2. Number of eggs 3. Number of eclosed offspring; measured for sympatric and allopatric crosses.	No significant differences in any trait between allopatric and sympatric crosses in either treatment.

* Gs. - number of generations of experimental evolution.

† Treatments for each study are listed from mildest to strongest sexual conflict.

‡ Sympatric crosses – between individuals from the same line. Allopatric crosses – between individuals from different lines within the same treatment.

§ Different direction opposite to what is predicted if sexual conflict drives reproductive isolation.

Conclusions of comparative studies are also equivocal. Species richness correlates with levels of sexual conflict in insects [37], but not in birds and mammals [38,39]. What is more, because differences in sexual conflict levels are necessarily confounded with differences in the strength of sexual selection, the number of species in insects may have been influenced by an effect of sexual selection on the adaptation rate [40,41,42,43] and/or the risk of extinction [43].

In conclusion our study shows that sexual conflict does not necessarily drive reproductive isolation between allopatric populations. After 35 and 45 generations of evolution under strong sexual conflict the bulb mite did not show either pre- or post-zygotic reproductive isolation. This conclusion is in line with predictions of theoretical models that incorporate the costs of sexually selected traits. Our results thus add to a growing number of examples questioning the role of speciation through sexual conflict as a ubiquitous phenomenon. However, more studies on a wider range of species are clearly needed as the hypothesis of reproductive isolation through sexual conflict has only been tested in a limited number of taxa.
